# Efficacy of Transcranial Direct Current Stimulation in the Treatment of Anorexia Nervosa—Interim Results from an Ongoing, Double-Blind, Randomized, Placebo-Controlled Clinical Trial

**DOI:** 10.3390/jcm14145040

**Published:** 2025-07-16

**Authors:** Zuzanna Rząd, Joanna Rog, Natalia Kajka, Maksymilian Seweryn, Jakub Patyk, Hanna Karakuła-Juchnowicz

**Affiliations:** 1 I Department of Psychiatry, Psychotherapy and Early Intervention in Lublin, Medical University of Lublin, 20-439 Lublin, Polandrog.joann@gmail.com (J.R.); natalia.kajka@umlub.pl (N.K.); 2 Students Research Group, I Department of Psychiatry, Psychotherapy and Early Intervention, Medical University of Lublin, 20-439 Lublin, Poland

**Keywords:** anorexia nervosa, eating disorders, brain stimulation, neuromodulation, tDCS, transcranial direct current stimulation, EAT-26

## Abstract

**Background/Objectives**: Anorexia nervosa (AN) is a severe disorder with limited treatment efficacy. This interim analysis aimed to assess the preliminary efficacy and safety of transcranial direct current stimulation (tDCS) in reducing core AN symptoms, stress, depression, low self-esteem, and BMI in adolescent females, to determine the rationale for continuing the study. **Methods**: A single-center, randomized, double-blind, placebo-controlled trial included 20 adolescent females with AN assigned to an active tDCS group *(n* = 10) or a sham group (*n* = 10). The intervention involved 30 sessions over three weeks, targeting the dorsolateral prefrontal cortex. Outcomes were assessed at baseline, post-treatment, and follow-up using the Eating Attitudes Test (EAT-26) for eating disorder symptoms, the Perceived Stress Scale (PSS-10) for stress, the Beck Depression Inventory (BDI) for depression, the Rosenberg Self-Esteem Scale (SES) for self-esteem, and body mass index (BMI) measurements. Safety and tolerability were assessed using the tDCS Side Effects Questionnaire. **Results**: Eating disorder symptoms significantly decreased in the active tDCS group at study end (*p* = 0.003) and follow-up (*p* = 0.02), while no significant changes were observed in the sham group. Although BMI increased more in the active group (13.78%) than in the sham group (7.31%), this difference was not statistically significant (*p* = 0.10). **Conclusions**: Adverse effects were mild and transient, with no serious safety concerns reported. Based on the results of this interim analysis, the study will proceed due to promising efficacy outcomes and good treatment tolerability.

## 1. Introduction

Anorexia nervosa (AN) is a severe psychiatric disorder characterized by an intense fear of gaining weight and a distorted body image, leading to restrictive eating behaviors, significant weight loss, and malnutrition. The prevalence of AN is greater in females (1.5%) than in males (0.3%), with a higher incidence in adolescents and young adults [1]. The disorder has one of the highest mortality rates among psychiatric conditions and is associated with dysfunctions of all organs and systems, including the gastrointestinal and endocrine [2]. AN is classified by the DSM-5 into two subtypes: the restricting subtype (AN-r), characterized primarily by reduced food intake and excessive physical activity; and the binge–purge subtype (AN-bp), involving episodes of binge eating and/or purging behaviors such as vomiting or laxative use [3]. These subtypes differ not only in behavior but also in personality traits and comorbidities: individuals with AN-bp tend to exhibit higher impulsivity and comorbid affective disorders, whereas those with AN-r show increased perseverance and are more likely to present with avoidant personality traits [4].

The underlying biological mechanisms of AN have yet to be fully elucidated. Current research suggests genetic predispositions and neurobiological abnormalities [5]. One of the key regions affected by AN is dorsolateral prefrontal cortex (DLPFC) [6]. Findings from both clinical and preclinical studies indicate that chronic starvation and excessive exercise, both common in AN, lead to structural and functional brain changes. Animal models such as activity-based anorexia (ABA) reproduce key features of AN, including compulsive hyperactivity, dysregulated reward processing, and cognitive rigidity. These effects have been linked to altered dopamine and serotonin signaling as well as glial loss in regions such as the DLPFC, striatum, and limbic system [7,8]. In humans, chronic undernutrition is associated with reduced gray and white matter volumes, diminished synaptic density, and neuroinflammatory responses [9], potentially contributing to persistent disturbances in executive function, mood regulation, and insight.

Treatment of AN encompasses various therapeutic approaches that operate through different mechanisms. Standard care for AN typically involves nutritional rehabilitation, psychotherapy, and pharmacological interventions, often in combination [10,11]. For adolescents, Family-Based Therapy (FBT) is currently the first-line treatment, exhibiting the highest efficacy among all behavioral therapies. Long-term outcomes for FBT indicate a full remission rate of 40–50%, with about 30% of patients achieving partial remission [12,13]. Cognitive–behavioral therapy (CBT) is a first choice for adults and is recommended for adolescents if FBT proves ineffective [14]. According to Danielsen et al., 60–70% of patients undergoing CBT achieve a BMI of 18.5% or higher within 20 weeks, with 40–50% maintaining their weight after one year [15]. Pharmacotherapy is generally not the primary treatment for AN. While selective serotonin reuptake inhibitors (SSRIs) are effective in treating comorbid conditions like depression and anxiety, they do not significantly aid in achieving a body mass index (BMI) above 18.5 kg/m^2^, limiting their direct impact on AN. Atypical antipsychotics, particularly olanzapine, have shown more promise by promoting weight gain and reducing obsessive symptoms [16].

The limited efficacy of traditional AN treatments prompts the search for new approach strategies. According to the neurobiological abnormalities, neuromodulatory and neurosurgical methods could appear promising. Among neuromodulatory methods, Deep Brain Stimulation (DBS), repetitive Transcranial Magnetic Stimulation (rTMS), and transcranial Direct Current Stimulation (tDCS) are particularly noteworthy [17]. tDCS is a non-invasive brain modulation technique that has already shown promising results as additional treatment in schizophrenia and depression [18]. tDCS involves applying a low electrical current to specific brain areas using electrodes placed on the scalp, and its therapeutic action involves modulating cortical excitability and increasing neuroplasticity [19]. The electrodes have opposite polarities, and the current flows between the anode (positive electrode) and the cathode (negative electrode), affecting neuron excitability in targeted brain areas and modulating cortical activity. Based on the polarity of the applied current, there are two types of stimulation. Anodal stimulation depolarizes neurons, making them more excitable, while cathodal stimulation hyperpolarizes neurons, inhibiting excitability [20]. By applying tDCS to the DLPFC, researchers aim to modulate the neurotransmitter system, which refers to processes influencing the activity and efficiency of signal transmission between neurons via neurotransmitters such as dopamine and serotonin. These neurotransmitters are closely linked to reward processing, mood regulation, and appetite control [3,21,22]. tDCS affects neurotransmission by altering cortical excitability, leading to increased or decreased release of specific neurotransmitters, thereby modulating neuronal function. By enhancing neuroplasticity and modulating these circuits, tDCS may offer a novel way to address the deep-seated cognitive and emotional components of AN that current behavioral therapies struggle to change [20].

The aim of the study was to conduct an interim assessment of the efficacy and safety of tDCS treatment in adolescent girls with AN. The collected data will allow us to evaluate the justification for continuing this research and the potential need to modify its parts. Our primary hypothesis is that tDCS applied to the DLPFC will significantly reduce eating disorder symptoms measured by Eating Attitudes Test—26 Item (EAT-26). Our secondary hypotheses are that participants receiving active tDCS will show a greater increase in BMI than those in the sham group by the end of the study and at follow-up, and active tDCS will lead to a significant reduction in depression symptoms measured by the Beck Depression Inventory (BDI), perceived stress levels measured by the Perceived Stress Scale (PSS-10), and improvements in self-esteem measured by The Rosenberg Self-Esteem Scale (SES) compared to the sham group.

## 2. Methods

### 2.1. Design and Intervention

The patients included in the study were randomly assigned to one of two subgroups, independently of examined variables, during the entry of the study: active group (AG) (tDCS group, n = 10) and sham group (SG) (placebo group, n = 10). Eligible patients were randomized in equal proportions between the AG and SG arms. A computerized random permuted block sequence generator (Urbaniak GC, Plous S. Research Randomizer; Version 4.0; 2013) was used to randomize active versus sham arms. The allocation to study groups was performed by a researcher not involved in the stimulation and assessment of patients. The detailed description of methods and procedures is available in the study protocol available at the link: https://www.frontiersin.org/journals/psychiatry/articles/10.3389/fpsyt.2024.1284675/full (accessed on 1 June 2025) [23]. Since the interim analysis was not part of the original protocol, no formal stopping rules were established. However, the research team reviewed the findings to evaluate the rationale for continuing the study in terms of efficacy and safety. Patients received either tDCS (with a current of 2 milliampere) or sham stimulation twice daily for 3 weeks on weekdays (totaling 30 sessions). Each session lasted 25 min and occurred with a two-hour break in between. Participants in the active tDCS group received stimulation to the DLPFC using two saline-soaked sponges positioned at F3 (anode) and F4 (cathode), following the International 10–20 system. Two weeks after the end of the stimulation, a follow-up visit took place. For the sham tDCS condition, the same setup was employed. When set to Sham, the device initially ramped up to the programmed intensity before reducing to a minimal test current of 25 microamps, which it maintained to verify contact quality throughout the session. At the session’s end, the device again ramped up to the programmed intensity and then back down to the test current. The ramp-up and ramp-down phases each lasted 30 s at the beginning and end of each sham session. For active stimulation, the device ramped up to the programmed intensity in 30 s and ramped down to the test current over 30 s at the end of the session. The Soterix Medical Inc. 1 × 1 tDCS mini-CT LTE device (model 1601-LTE) was used for all stimulations. The study was designed according to the guidelines of the Recommendations for Interventional Trials (SPIRIT, 2013) [24], and received a positive opinion of the Bioethics Committee at the Medical University of Lublin (ID: KE-0254/24/01/2022). The study was registered in the clinicaltrials.gov database (study ID: NCT05814458). The study was conducted by the principles of the Declaration of Helsinki [25] and the principles of Good Clinical Practice [26].

### 2.2. Trial Oversight

The study was designed collaboratively by academic researchers. Oversight was provided by an independent external data and safety monitor (JR), who was not affiliated with the researchers’ institution. The monitor, who had a background in dietetics along with extensive experience in statistical analysis of biomedical data and non-commercial clinical trials, conducted regular safety assessments. To ensure the integrity of blinding, JR had no involvement in patient-related procedures or data collection and was the only individual with access to a subset of unblinded results. Meanwhile, the investigators remained blinded throughout the study.

### 2.3. Participants

The study participants were patients hospitalized for AN at the 1st Department of Psychiatry, Psychotherapy, and Early Intervention, Lublin, Poland. The inclusion criteria were as follows: Written informed consent required before study procedures; for minors, consent from a parent or guardian. Female patients aged 10–30 years, meeting DSM-5 criteria for Anorexia Nervosa (AN) and with BMI ≤ 17.5 kg/m^2^ [27]. Additionally, the only permitted form of psychopharmacotherapy was a stable dose of antidepressants, such as SSRIs, serotonin norepinephrine reuptake inhibitors (SNRIs), or tricyclic antidepressants, maintained for at least one month before the screening visit. Exclusion criteria were as follows: lack of informed consent; male gender; diagnosis of neurological or organic diseases, such as epilepsy; contraindications to tDCS (e.g., presence of pacemakers, metal implants in the head area); the presence of any psychiatric comorbidities except for mood disorders recognized as a consequence following AN; pregnancy or plans to become pregnant; and any changes in psychopharmacotherapy during the study.

### 2.4. Timeline

This study consists of four visits:

V0: Screening visit to screen and enroll eligible patients into the study (1 to 2 days before the V1).
V1: Baseline visit (1 day before intervention) to randomize patients and conduct assessments.
V2: End of the stimulation, up to 3 days after the end of the intervention period, after three weeks (±1 day) from V1.
V3: Follow-up visit to complete all procedures, after 5 weeks (±7 days) from V1.

### 2.5. Outcome Measures

The full set of assessment methods used in the study is detailed in the study protocol. To assess the necessity of continuing the study, only the following criteria were included in the work:

- For sociodemographic data: Self-prepared personal questionnaire. The survey covered personal data, medical history, information on menstruation, and used pharmacotherapy.
- For assessing eating behaviors: EAT-26 part A. Standardized test for the detection of symptoms of eating disorders. It is used for screening the population at risk of anorexia, bulimia, and obesity. In addition to the global score, it is also possible to analyze three EAT-26 domains, such as (1) Dietary Behavior, (2) Bulimia and Food Preoccupation, and (3) Oral Control [28].
- For psychological data: The Beck Depression Inventory (BDI) it is a self-assessment tool helpful in screening the diagnosis of depression and measuring its severity [29]. The Perceived Stress Scale (PSS-10) is used to measure stress based on subjective feelings related to problems and personal events, behavior, and coping with stress [30]. The Rosenberg Self-Esteem Scale (SES) is a tool for assessing the level of general self-esteem—a relatively stable disposition understood as a conscious attitude (positive or negative) towards the Self [31].
- For anthropometric data: Body weight and body mass index (BMI) were measured.
- For the safety assessment: tThe tDCS Side Effects Questionnaire by Brunoni et al. was utilized [32].

### 2.6. Statistical Analysis

The statistical analysis was carried out with STATISTICA 13.0 computer software. The distribution of continuous variables was verified with the Shapiro–Wilk test. The descriptive statistics were reported as means and standard deviations (SDs) (ex-Gaussian distribution) or median and range (no ex-Gaussian distributions) for continuous variables, as well as percentages for qualitative variables. To assess the effect of tDCS on outcomes examined at three time points, a mixed-design ANOVA with repeated measurements with post hoc Bonferroni test was performed. To assess the effect of tDCS between groups at two time points, Student’s *t*-test for dependent variables (ex-Gaussian distribution) and the Wilcoxon test (no ex-Gaussian distribution) were carried out. To determine differences between groups at V2 and V3, their baseline characteristics, Student’s *t*-test (ex-Gaussian distribution) and the U-Mann–Whitney test (no ex-Gaussian distribution) were carried out. The level of significance was set as *p* < 0.05.

## 3. Results

The baseline characteristics of patients are depicted in Table 1. There were no differences between groups in the baseline demographic and clinical characteristics.

**Effect of tDCS on BMI and psychopathological symptoms**

At baseline, there were no significant differences between the active group (AG) and the sham group (SG) in the psychopathology of eating disorders or comorbid symptoms (*p* > 0.05). The SG had similar severity of eating disorder symptoms in all timelines (*p* > 0.05). In the AG, the severity of eating disorder symptoms decreased at the end of the study (*p* = 0.007) and follow-up (*p* = 0.044) compared to baseline (18 (5–53); 11 (1–46) vs. 49.5 (5–59), respectively (see Table 2). We found the interaction between treatment and time on perceived stress symptoms (*p* = 0.04) and time effect on self-esteem (*p* = 0.11). However, after post hoc analysis, no significant difference was found (see Table 3 and Table 4). We found differences in BMI at the end of the study and follow-up compared to baseline in the AG (*p* < 0.001; 16.12 ± 1.38, 16.57 ± 1.22 vs. 14.81 ± 1) and SG (*p* = 0.04; 16.38 ± 1.24 and *p* = 0.004; 16.57 ± 1.11 vs. 15.27 ± 1.09) (see Table 5). In both groups, we did not find any changes in depressive symptoms between baseline, end of the study, and follow-up (*p* > 0.05) (see Table 6).

We did not find differences in total scores of EAT-26, PSS-10, SES at the end of the study and follow-up between AG and SG (*p* > 0.05) as shown in Table 2, Table 3 and Table 4.

Eating disorder symptoms significantly decreased in the active tDCS group at study end (*p* = 0.003) and follow-up (*p* = 0.02), while no significant changes were observed in the sham group. In addition, a separate analysis of individual EAT-26 subscales in the AG was conducted to further explore specific symptom domains. According to the repeated measures ANOVA, no statistically significant group × time interaction was found in the Dieting subscale (F = 1.02, *p* = 0.149). However, post hoc analysis revealed significant within-group improvements in the AG between baseline and both the end of the study (*p* = 0.001) and the follow-up (*p* = 0.0008). A statistically significant group × time interaction was observed in the Oral Control subscale (F = 4.82, *p* = 0.0147), while a trend toward significance was noted in the Bulimia and Food Preoccupation subscale (F = 5.31, *p* = 0.10). Post hoc Bonferroni-corrected comparisons in the AG indicated the following significant within-group changes: I. In the Bulimia and Food Preoccupation subscale: between baseline and both the end of the study (*p* = 0.0049) and the follow-up (*p* = 0.0003). II. In the Oral Control subscale: between baseline and both the end of the study (*p* = 0.0001) and the follow-up (*p* = 0.000003).

BMI increased in both groups between V1 and V3 but was higher in the active tDCS group (13.78%) than in the sham group (7.31%), though the difference was not statistically significant (*p* = 0.10) (Table 5).

Although a significant main effect of time was observed for BDI scores (F = 4.52, *p* = 0.019), indicating an overall reduction in depressive symptoms, the group × time interaction was not statistically significant (F = 0.35, *p* = 0.071). This suggests that the observed improvement in depression severity cannot be specifically attributed to the tDCS intervention (Table 6).

## 4. Safety

To monitor the safety and tolerability of the neurostimulation, the tDCS Side Effects Questionnaire by Brunoni et al. was utilized [32]. This questionnaire was designed to standardize and improve the reporting of adverse events in clinical trials involving tDCS, aiming to enhance the understanding of its safety and tolerability. In our study, 4 (40%) girls from the active group and 2 (20%) from the control group experienced adverse events, including slight headaches and dizziness. In the active group, 3 girls (30%) reported headaches, 2 girls (20%) experienced dizziness, and 1 girl (10%) experienced both symptoms simultaneously. In the control group, 1 girl (10%) reported a headache, and 1 girl (10%) experienced dizziness. These events were classified as Grade 1 (mild) according to the Common Terminology Criteria for Adverse Events (CTCAE) and consistent with the side effects of tDCS described in the literature [33]. In the active group, all participants showed mild, localized redness at the treatment site, likely at the electrode placement areas, which disappeared quickly within an hour and did not cause pain. The symptoms appeared shortly after the intervention and lasted only briefly, with all participants fully recovering within a few hours. None of the adverse events were serious, as they did not require hospitalization or result in any long-term effects. No specific treatment was needed, as the symptoms were not severe enough to cause significant discomfort or disability. The adverse events were probably related to the treatment, though they remained within the expected range based on prior clinical findings. All participants fully recovered, with no lasting effects.

## 5. Discussion

The results presented are preliminary data from a single-center, randomized, double-blind study; they were unblinded by an independent data monitor and analyzed to assess whether the continuation of the study was still scientifically justified. This analysis aimed to assess the efficacy and safety of 30 sessions of tDCS stimulation on the left DLPFC in patients AN. The primary analysis showed a positive impact of tDCS treatment on EAT-26 scores. Eating disorder symptoms showed a significant reduction in the active tDCS group both at the end of the intervention and at follow-up, whereas no meaningful changes were observed in the sham group. Based on the results observed in the AG, it appears that the intervention may exert its effects in a domain-specific manner. Significant improvements were noted particularly in the Oral Control and Bulimia and Food Preoccupation subscales, which may reflect enhanced self-regulatory capacity and reduced compulsive or intrusive food-related thoughts. These changes suggest that tDCS targeting the DLPFC may modulate neural circuits involved in behavioral inhibition, impulse control, and emotional regulation. The lack of significant interaction effects in the Dieting subscale, despite within-group improvement, could indicate that general cognitive restraint and body image dissatisfaction are less responsive to short-term neuromodulation and may require complementary psychotherapeutic strategies to address more entrenched beliefs and attitudes. These findings suggest that tDCS may exert differential effects across specific domains of eating disorder psychopathology, particularly in areas related to cognitive restraint and behavioral control over food intake.

Collectively, these findings support the hypothesis that tDCS may facilitate symptom reduction in specific components of eating disorder psychopathology, particularly those associated with compulsive behaviors and impaired control. Further data collection is required to confirm these preliminary observations.

The secondary analysis did not show a significant effect on weight recovery in patients with AN. The BMI values increased from V1 to V3 in both groups, which could be attributed to standardized realimentation procedures during hospitalization and strict control by medical staff over the patients’ eating habits. These might be confounding variables and need to be considered in further analysis of this study. Based on these observations, a future extension of the study design could include an additional control group of inpatients receiving only treatment-as-usual, without tDCS or sham.

Our study provides preliminary insights into the efficacy of tDCS in treating ED behaviors, stress, and BMI. We did not show significant changes in depression symptoms between the AG and the SG, which are frequently observed over the course of AN [24,34]. The literature suggests that tDCS may improve depressive symptoms [25,35], leading us to anticipate an improvement in BDI scores for the AG. However, due to the limited sample size and ongoing data collection, definitive conclusions cannot yet be drawn. Despite a trend toward reduced stress in the AG, the between-group differences were not statistically significant (*p* = 0.04 for interaction effect). This suggests that while tDCS may influence stress regulation, the effect size may be small, requiring further validation in larger samples. This aligns with previous findings that DLPFC stimulation may influence the hypothalamic–pituitary–adrenal (HPA) axis, potentially lowering cortisol levels and improving the body’s response to stress. Despite a trend toward reduced stress in the AG, the between-group differences were not statistically significant (*p* = 0.04 for interaction effect). This suggests that while tDCS may influence stress regulation, the effect size may be small, requiring further validation in larger samples. Future studies should consider larger sample sizes and additional biomarkers of stress regulation, such as salivary cortisol, to further investigate these effects. Similarly, while self-esteem (SES) scores showed an increasing trend in the AG, the differences were not statistically significant. Given that self-esteem is a multifaceted construct influenced by long-term cognitive and emotional factors, it is possible that the short duration of the study was insufficient to observe significant improvements. Additionally, the relationship between weight restoration and self-esteem in AN patients is complex, as weight gain can sometimes be perceived negatively by patients despite being a key treatment goal. Our findings support previous reports on the safety of tDCS in the pediatric population, where the predominant side effect was mild itching, and most sessions were conducted without reported adverse events [36]. Considering the high safety of the intervention and the promising preliminary results, and in accordance with the published protocol, the study will continue to further assess the efficacy of tDCS in the treatment of anorexia [23]. Further analysis with a larger sample will provide a more comprehensive understanding of the long-term effects and potential clinical applications of this therapy.

Our study has several limitations that should be taken into account when interpreting the findings. First, it is important to note that the current results are based on interim data and should be viewed as preliminary rather than conclusive. Second, the inclusion of a broader study design with an additional control group receiving only treatment-as-usual—without active or sham tDCS—would strengthen future research by enabling clearer attribution of treatment effects. Third, the relatively broad age range of participants may contribute to variability in outcomes, as neurodevelopmental stage and brain plasticity can significantly influence responsiveness to neuromodulatory interventions. Fourth, body mass index (BMI) was used in the current analysis; however, BMI z-scores or percentiles are more appropriate for adolescents and will be incorporated in future analyses to ensure more accurate age-adjusted comparisons. The final analysis of the full dataset will offer a more comprehensive assessment of the intervention’s efficacy.

## Figures and Tables

**Table 1:** Baseline characteristics of patients.

	AG	SG	Differences (AG vs. SG)	
			Z-Value/χ2-Value	*p*-Value
Age, years	14.5 (13–23)	15 (13–27)	0.54	0.58
Subtype *Restricting AN* *Binge–purge AN*	8 (80%) 2 (20%)	7 (70%) 3 (30%)	0.27	0.61
Duration of illness (months)	39.90 (10–180)	25 (6–120)	1.10	0.29
Menstrual status *Primary amenorrhea* *Secondary amenorrhea* *Normal menstrual cycle*	0 (0%) 10 (100%) 0 (0%)	1 (10%) 8 (80%) 1 (10%)	2.22	0.33
Medication intake	5 (50%)	7 (70%)	1.81	0.26
BMI (kg/m^2^)	14.81 ± 1.09	15.27 ± 1.00	1.50	0.33

Abbreviations: AN—anorexia nervosa, BMI—body mass index, AG—active group, SG—sham group.

**Table 2:** Changes in the EAT-26 after tDCS stimulation.

Group	EAT-26 I	EAT-26 II	EAT-26 III	Treatment (F)	Time (F)	Treatment xTime (F)	Differences
Active Group	49.5 (5–59)	18 (5–53)	11 (1–46)	0.02 (*p* = 0.88)	6.35 (*p* = 0.005) *	2.79 (*p* = 0.076)	I vs. II *p* = 0.007 I vs. III *p* = 0.044
Sham Group	28.5 (0–67)	25.5 (0–56)	33 (1–50)				N/S

Abbreviations: EAT-26—Eating Attitudes Test; * Treatment—active/placebo; Time—baseline, end of the study, follow-up; F—statistic from mixed-design ANOVA with repeated measures; N/S—not significant.

**Table 3:** Changes in the PSS-10 after tDCS stimulation.

Group	PSS-10 I	PSS-10 II	PSS-10 III	Treatment (F)	Time (F)	Treatment xTime (F)	Differences
Active	28 ± 7.64	22.3 ± 7.82	21.56 ± 6.97	0.02 (*p* = 0.90)	2.22 (*p* = 0.125)	3.50 (*p* = 0.04) *	N/S
Sham	23.2 ± 9.75	22 ± 7.35	24.67 ± 7.45				N/S

Abbreviations: PSS-10—Perceived Stress Scale; * Treatment—active/placebo; Time—baseline, end of the study, follow-up; F—statistic from mixed-design ANOVA with repeated measures; N/S—not significant.

**Table 4:** Changes in the SES after tDCS stimulation.

Group	SES I	SES II	SES III	Treatment (F)	Time (F)	Treatment xTime (F)	Differences
Active	20.7 ± 4.85	21.3 ± 3.65	22.78 ± 4.12	2.31 (*p* = 0.15)	5.25 (*p* = 0.011) *	0.75 (*p* = 0.048) **	N/S
Sham	24 ± 6.11	23.78 ± 4.82	26.11 ± 4.04				N/S

Abbreviations: SES—Self-Esteem Scale; * Treatment—active/placebo; ** Significant interaction effect, but no significant pairwise post hoc comparisons (*p* > 0.05); Time—baseline, end of the study, follow-up; F—statistic from mixed-design ANOVA with repeated measures; N/S—not significant.

**Table 5:** Changes in the BMI after tDCS stimulation.

Group	BMI I (kg/m^2^)	BMI II (kg/m^2^)	BMI III (kg/m^2^)	Treatment (F)	Time (F)	Treatment xTime (F)	Differences
Active	14.81 ± 1.0	16.12 ± 1.38	16.57 ± 1.22	0.78 (*p* = 0.40)	43.45 (*p* < 0.001) *	3.51 (*p* = 0.046) *	I vs. II *p* < 0.001 I vs. III *p* < 0.001
Sham	15.27 ± 1.09	16.38 ± 1.24	16.57 ± 1.11				I vs. II *p* = 0.04 I vs. III *p* = 0.004

Abbreviations: BMI—Body Mass Index; * Treatment—active/placebo; Time—baseline; end of the study, follow-up; F—statistic from mixed-design ANOVA with repeated measures.

**Table 6:** Changes in the BDI after tDCS stimulation.

Group	BDI I	BDI II	BDI III	Treatment (F)	Time (F)	Treatment xTime (F)	Differences
Active	24.11 ± 10.51	20.7 ± 12.18	21.22 ± 13.4	0.25 (*p* = 0.62)	4.52 (*p* = 0.019) *	0.35 (*p* = 0.071)	N/S
Sham	20.7 ± 11.3	17.5 ± 9.4	15.89 ± 8.36				N/S

Abbreviations: BDI—Beck Depression Inventory; N/S—not significant; * Treatment—active/placebo; Time—baseline, end of the study, follow-up; F—statistic from mixed-design ANOVA with repeated measures.

## Data Availability

The data presented in this study are available on request from the corresponding author. The data are not publicly available due to privacy or ethical restrictions.

## Abbreviations

The following abbreviations are used in this manuscript:

AN	Anorexia Nervosa
tDCS	Transcranial Direct Current Stimulation
DLPFC	Dorsolateral Prefrontal Cortex
BMI	Body Mass Index
EAT-26	Eating Attitudes Test—26 Item
PSS-10	Perceived Stress Scale—10 Item
BDI	Beck Depression Inventory
SES	Rosenberg Self-Esteem Scale
AG	Active Group
SG	Sham Group
CBT	Cognitive Behavioral Therapy
FBT	Family-Based Therapy
SSRI	Selective Serotonin Reuptake Inhibitor
SNRI	Serotonin Norepinephrine Reuptake Inhibitor
DBS	Deep Brain Stimulation
rTMS	Repetitive Transcranial Magnetic Stimulation
SPIRIT	Standard Protocol Items: Recommendations for Interventional Trials
